# Vascular endothelial growth factor, platelet-derived endothelial cell growth factor and angiogenesis in non-small-cell lung cancer

**DOI:** 10.1054/bjoc.1999.1129

**Published:** 2000-03-21

**Authors:** K J O'Byrne, M I Koukourakis, A Giatromanolaki, G Cox, H Turley, W P Steward, K Gatter, A L Harris

**Affiliations:** University Department of Oncology, Leicester Royal Infirmary, Leicester, LE1 5WW, UK; Department of Radiotherapy & Oncology, Laboratory of Cancer Biology, University Hospital of Iraklion, Iraklion, Crete, 71110, Greece; Cellular Science and Imperial Cancer Research Fund Medical Oncology Unit, Oxford Radcliffe Hospitals Trust, Oxford, OX3 7LJ, UK

**Keywords:** vascular endothelial growth factor, platelet-derived endothelial cell growth factor, angiogenesis, non-small cell lung cancer

## Abstract

High microvessel density, an indirect measure of angiogenesis, has been shown to correlate with increased tumour size, lymph node involvement and poor prognosis in non-small-cell lung cancer (NSCLC). Tumour cell vascular endothelial growth factor (VEGF) and platelet-derived endothelial cell growth factor (PD-ECGF) expression correlate with angiogenesis and a poor outcome in this disease. In a retrospective study VEGF and PD-ECGF expression and microvessel density were evaluated immunohistochemically in surgically resected specimens (T1–3, N0–2) from 223 patients with operable NSCLC using the VG1, P-GF.44C and JC70 monoclonal antibodies respectively. High VEGF immunoreactivity was seen in 104 (46.6%) and PD-ECGF in 72 (32.3%) cases and both were associated with high vascular grade tumours (*P* = 0.009 and *P* = 0.05 respectively). Linear regression analysis revealed a weak positive correlation between VEGF and PD-ECGF expression in cancer cells (*r* = 0.21;*P* = 0.002). Co-expression of VEGF and PD-ECGF was not associated with a higher microvessel density than VEGF or PD-ECGF only expressing tumours. Furthermore a proportion of high vascular grade tumours expressed neither growth factor. Univariate analysis revealed tumour size, nodal status, microvessel density and VEGF and PD-ECGF expression as significant prognostic factors. Tumour size (*P*< 0.02) and microvessel density (*P*< 0.04) remained significant on multivariate analysis. In conclusion, VEGF and PD-ECGF are important angiogenic growth factors and have prognostic significance in NSCLC. Furthermore the study underlines the prognostic significance of microvessel density in operable NSCLC. © 2000 Cancer Research Campaign


*British Journal of Cancer* (2000) **82**(8), 1427–1432
© 2000 Cancer Research Campaign
DOI: 10.1054/ bjoc.1999.1129, available online at http://www.idealibrary.com on IDEAL®

### Vascular endothelial growth factor, platelet-derived endothelial cell growth factor and angiogenesis in non-small-cell lung cancer

**KJ O'Byrne[1], MI Koukourakis[2], A Giatromanolaki[2], G Cox[1], H Turley[3], WP Steward[1], K Gatter[3] and AL Harris[4]**

[1]University Department of Oncology, Leicester Royal Infirmary, Leicester LE1 5WW, UK; [2]Department of Radiotherapy & Oncology, Laboratory of Cancer Biology, University Hospital of Iraklion, Iraklion 71110, Crete, Greece; Department of [3]Cellular Science and [4]Imperial Cancer Research Fund Medical Oncology Unit, Oxford Radcliffe Hospitals Trust, Oxford OX3 7LJ, UK

**Summary** High microvessel density, an indirect measure of angiogenesis, has been shown to correlate with increased tumour size, lymph node involvement and poor prognosis in non-small-cell lung cancer (NSCLC). Tumour cell vascular endothelial growth factor (VEGF) and platelet-derived endothelial cell growth factor (PD-ECGF) expression correlate with angiogenesis and a poor outcome in this disease. In a retrospective study VEGF and PD-ECGF expression and microvessel density were evaluated immunohistochemically in surgically resected specimens (T1–3, N0–2) from 223 patients with operable NSCLC using the VG1, P-GF.44C and JC70 monoclonal antibodies respectively. High VEGF immunoreactivity was seen in 104 (46.6%) and PD-ECGF in 72 (32.3%) cases and both were associated with high vascular grade tumours ($P = 0.009$ and $P = 0.05$ respectively). Linear regression analysis revealed a weak positive correlation between VEGF and PD-ECGF expression in cancer cells ($r = 0.21$; $P = 0.002$). Co-expression of VEGF and PD-ECGF was not associated with a higher microvessel density than VEGF or PD-ECGF only expressing tumours. Furthermore a proportion of high vascular grade tumours expressed neither growth factor. Univariate analysis revealed tumour size, nodal status, microvessel density and VEGF and PD-ECGF expression as significant prognostic factors. Tumour size ($P < 0.02$) and microvessel density ($P < 0.04$) remained significant on multivariate analysis. In conclusion, VEGF and PD-ECGF are important angiogenic growth factors and have prognostic significance in NSCLC. Furthermore the study underlines the prognostic significance of microvessel density in operable NSCLC. © 2000 Cancer Research Campaign

**Keywords:** vascular endothelial growth factor; platelet-derived endothelial cell growth factor; angiogenesis; non-small cell lung cancer

Angiogenesis is essential for tumour growth beyond 1–2 mm in diameter and plays a central role in the metastatic spread of malignant disease. Previous retrospective and prospective studies in non-small-cell lung cancer (NSCLC) have clearly demonstrated that angiogenesis, assessed by microvessel counting, is an important prognostic factor in operable NSCLC, high microvessel counts being associated with disease spread and a poor survival (Giatromanolaki et al, 1996; Fontanini et al, 1997).

Vascular endothelial growth factor (VEGF) is a potent and specific endothelial cell mitogenic and migratory, and vascular permeability, factor. Although produced from the same gene at least six different isoforms of 121, 145, 148, 165, 189 and 206 amino acids respectively have been identified (Ferrara and Davis-Smyth, 1997; Whittle et al, 1999). In the normal lung, VEGF may be expressed by bronchiolar and differentiated columnar epithelium and alveolar macrophages. Stromal fibroblasts and macrophages are only occasionally positive. In NSCLC tumours, VEGF-expressing blood vessels are identified in > 50% of cases. However, VEGF immunoreactivity is seen in < 10% of infiltrating lymphocytes (Giatromanolaki et al, 1998; Turley et al, 1998). Recent studies indicate that VEGF expression correlates with high microvessel counts and a poor prognosis in NSCLC (Volm et al, 1996; Giatromanolaki et al, 1998; Oshika et al, 1998; Fontanini et al, 1999).

Platelet-derived endothelial cell growth factor (PD-ECGF) is a non-heparin binding angiogenic factor initially isolated from platelets. Subsequent studies showed that PD-ECGF is a 90 kDa homodimer and is thymidine phosphorylase (TP) (Ishikawa et al, 1989; Moghaddam and Bicknell, 1992). Transfection of the PD-ECGF gene into transformed fibroblasts in nude mice results in neo-angiogenesis (Ishikawa et al, 1989). Stimulation of endothelial cell migration in vitro and enhancement of tumour growth in vivo have also been reported (Moghaddam et al, 1995). The precise mechanism by which PD-ECGF promotes angiogenesis is unclear. PD-ECGF hydrolyses thymidine to thymine and 2′-deoxy-D-ribose-1-phosphate. 2′-deoxy-D-ribose-1 phophate is dephosphorylated to 2′ deoxy-D-ribose which is angiogenic in the chicken-chorioallantoic membrane assay (Moghaddam et al, 1995). In normal lung, PD-ECGF expression is invariably seen in alveolar macrophages. Bronchiolar epithelium occasionally shows positive immunoreactivity. Bronchial basal and differentiated columnar cells are weakly positive. Weak immunoreactivity is also seen in stromal fibroblasts (Giatromanolaki et al, 1997). Recent studies indicate that tumour cell, but not stromal cell, PD-ECGF immunoreactivity correlates with angiogenesis and prognosis in NSCLC, high expression being associated with angiogenesis and a poor outcome (Koukourakis et al, 1998).

The co-expression of VEGF and PD-ECGF may enhance neovascularization in solid tumours (Toi et al, 1995; Maeda et al, 1997; Ikeda et al, 1999). In this retrospective study we report our

*Received 29 March 1999*
*Revised 11 November 1999*
*Accepted 11 November 1999*

*Correspondence to:* KJ O'Byrne findings on VEGF and PD-ECGF expression in an expanded series of 223 cases of NSCLC including T3 and N2 tumours. In particular the study evaluates the impact of the co-expression of these factors on angiogenesis and overall survival.

### MATERIALS AND METHODS

### Angiogenesis assessment

The JC70 monoclonal antibody (Dako) recognizing CD31 (platelet/endothelial cell adhesion molecule; PECAM-1) was used for microvessel staining on 5-μm paraffin-embedded tissue sections using the alkaline phosphatase/anti-alkaline phosphatase (APAAP) procedure as previously described (Giatromanolaki et al, 1996). Microvessel counting was used for angiogenesis assessment. The areas of the highest vascularization were chosen at low power (×100) and microvessel counting performed on three chosen ×250 fields to establish the highest density within the tumour. The microvessel score was the sum of the vessel counts obtained in these three fields. Microvessels adjacent to normal lung were excluded from the appraisal. Vessels with a clearly defined lumen or well defined linear vessel shape, but no single endothelial cells, were counted. Microvessel scores ≥ 75 defined high vascular grade disease, and < 75, low vascular grade tumours. This cut-off point was based on a previous study where microvessel scores of 75 or higher defined a group of cases with the highest death rate as compared with other cut-off points (Giatromanolaki et al, 1998).

### VEGF immunohistochemistry

VEGF immunoreactivity was evaluated employing the VG1 monoclonal antibody and the horseradish peroxidase technique as previously described (Giatromanolaki et al, 1998; Turley et al, 1998). The VG1 monoclonal antibody, which recognizes the 121, 165 and 189 isoforms of VEGF, was raised using recombinant VEGF 189 protein and the specificity of the antibody was confirmed using COS cells transfected with cDNA coding for VEGF 121, 165 and 189 protein and by Western blot studies. Sections were dewaxed and incubated in 0.5% hydrogen peroxide ($H_2O_2$) in methanol for 30 min. After microwaving and washing in phosphate-buffered saline (PBS), sections were incubated with the primary antibody for 60 min. After washing in PBS for 5 min, sections were incubated with goat anti-mouse immunoglobulin (1:2000) for 30 min (Dako, UK), washed again with PBS for 5 min and incubated with rabbit anti-goat immunoglobulin (1:100) for 30 min. The peroxidase reaction was developed using diaminobenzidine (Sigma Fast tablets) as chromogen and sections were counterstained with haematoxylin. Normal rabbit immunoglobulin-G was substituted for primary antibody as the negative control (same concentration as the test antibody). Taking into account the extent of positive staining, we divided our cases into two categories: low reactivity (0–70% positive cells) and high immunoreactivity (> 70% positive cells).

### PD-ECGF immunoreactivity

PD-ECGF expression in NSCLC tissue sections was assessed with the P-GF.44C monoclonal antibody as previously described (Giatromanolaki et al, 1997; Koukourakis et al, 1998). Staining was performed using the streptavidin-biotin-peroxidase (Dako, UK) technique. Sections were dewaxed and incubated in 0.5% $H_2O_2$ in methanol for 30 min. After washing in Tris-buffered saline (TBS), sections were incubated in normal human serum (1:10) for 20 min. Sections were then washed with TBS for 5 min and incubated with the undiluted primary antibody for 30 min. After washing in TBS for 5 min, sections were incubated with biotinylated goat anti-mouse immunoglobulin (1:200) for 30 min (Dako, UK). After incubation with the streptavidin–biotin complex–horseradish peroxidase (Dako, UK) for 30 min, the peroxidase reaction was developed using diaminobenzidine (Sigma Fast tablets) as chromogen, and sections were counter-stained with haematoxylin. Normal rabbit immunoglobulin-G was substituted for primary antibody as the negative control. Alveolar macrophages were used as a positive internal control (Giatromanolaki et al, 1997).

Tumour cell component was assessed for PD-ECGF expression by the intensity and extent of staining (Figure 1D). Two staining patterns of PD-ECGF immunoreactivity were considered: low reactivity (< 50% of cancer cells stained or diffuse weak reactivity) and high reactivity (strong staining in > 50% cells).

### Intra- and interobserver variability

In previous reports we have demonstrated that intraobserver variability was minimal for vascular grade and PD-ECGF with the second assessment correlating with the first for all observers ($r = 0.91$, $P < 0.006$ and $r = 0.96$, $P < 0.001$ respectively). Similarly the interobserver variability of three investigators was low ($r = 0.94$, $P < 0.001$ for vascular grade and $r = 0.91$, $P < 0.008$ for PD-ECGF) (Koukourakis et al, 1998). We made a similar observation for interobserver variability for VEGF ($r > 0.93$, $P < 0.0001$) (Giatromanolaki et al, 1998). Therefore intra- and interobserver variability were not formally assessed in this study.

### Statistics

Statistical analysis and graphic presentation were performed using the Stata 3.1 (Stata Corporation, Texas, USA) and the GraphPad Prism 2.01 package. The unpaired two-tailed *t*-test or Fisher's exact test was employed to test for relationships between categorical tumour variables as appropriate. Linear regression analysis was used to assess correlation between continuous variables. Survival curves were plotted using the method of Kaplan–Meier, and the log-rank test was used to determine statistical differences between life tables. A Cox proportional hazard model was used to assess the effects of patient and tumour variables on overall survival. A *P*-value < 0.05 was considered significant.

### RESULTS

Of the 223 tumours studied 156 were squamous cell carcinomas and 67 adenocarcinomas. Forty-three patients had stage 1a, 77 stage 1b, 22 stage IIa, 52 stage IIb and 29 stage IIIa NSCLC tumours. Survival data was available for 183 patients, patients dying within 60 days of surgery being excluded to avoid bias from peri-operative death. The median follow-up at the time of analysis for patients alive was 3.5 years (range 1.5–7 years).

 © 2000 Cancer Research Campaign

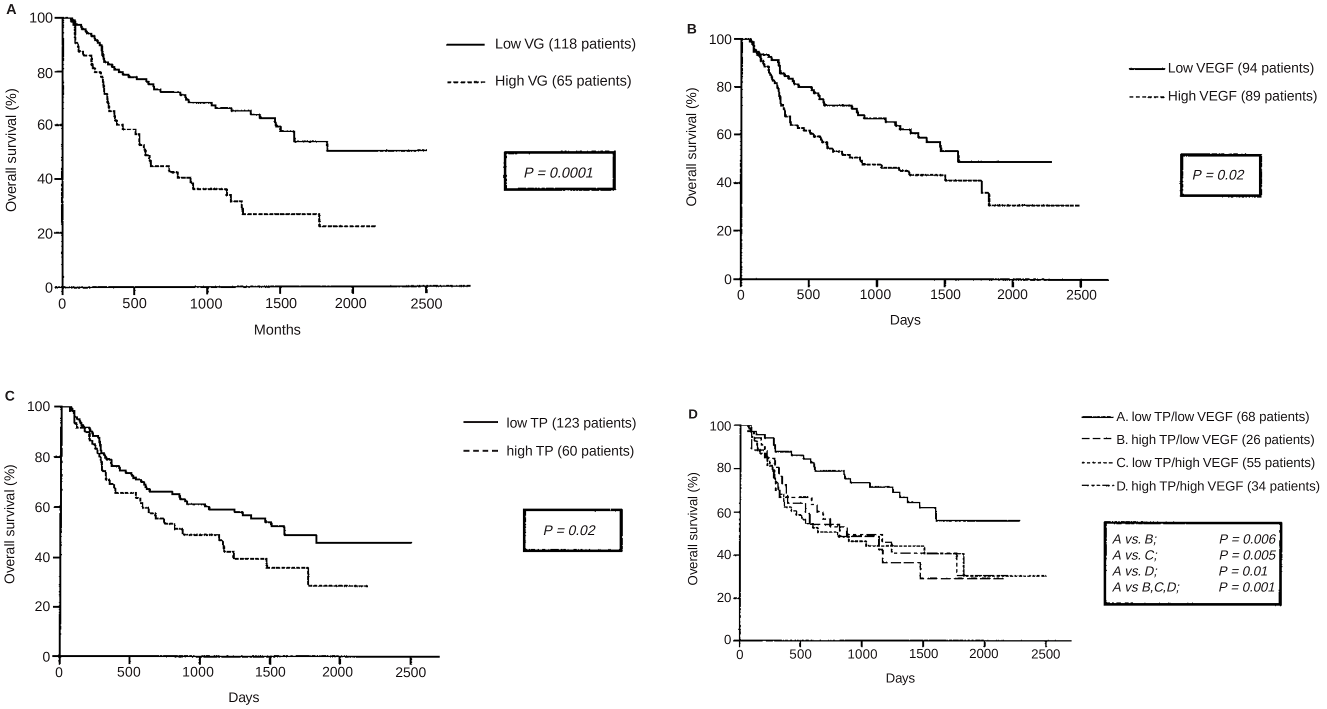

**Figure 1** Kalpan–Meier's survival curves. (**A**) VG = vascular grade: high vascular grade vs low vascular grade disease; (**B**). VEGF = vascular endothelial growth factor: high VEGF vs low VEGF immunoreactive tumours; (**C**). TP = thymidine phosphorylase which is PD-ECGF: high TP (PD-ECGF) *vs* low TP (PD-ECGF) immunoreactive tumours; (**D**) TP(PD-ECGF)–/VEGF– *vs* TP(PD-ECGF)+/VEGF– *vs* TP(PD-ECGF) ECGF)–/VEGF+ *vs* TP(PD-ECGF)+/ VEGF+ immunoreactive tumour subsets

**Table 1** Correlation of vascular grade with tumour parameters in 223 patients with stage I–IIIA non-small-cell lung cancer

| | | Microvessel score | | |
|---|---|---|---|---|
| **Parameters** | **No. of patients** | **Low** | **High** | ***P*-value** |
| Histology | | | | |
| Squamous cell | 156 | 94 | 62 | 0.44 |
| Adenocarcinoma | 67 | 44 | 23 | |
| T-stage | | | | |
| T1 | 68 | 46 | 22 | |
| T2 | 135 | 86 | 49 | 0.007 |
| T3 | 20 | 6 | 14 | |
| N-stage | | | | |
| N0 | 127 | 92 | 35 | |
| N1 | 77 | 39 | 38 | 0.0005 |
| N2 | 19 | 7 | 12 | |
| Grade | | | | |
| 1/2 | 109 | 70 | 39 | 0.44 |
| 3 | 114 | 68 | 46 | |
| VEGF | | | | |
| Negative | 119 | 83 | 36 | 0.009 |
| Positive | 104 | 55 | 49 | |
| PD-ECGF | | | | |
| Negative | 151 | 100 | 51 | 0.05 |
| Positive | 72 | 38 | 34 | |

### Vascular grade, VEGF and PD-ECGF

Eighty-nine of the 223 tumours (39.9%) were of high vascular grade. High microvessel counts were associated with advanced T-stage ($P = 0.007$) and node-positive ($P = 0.0005$) disease (Table 1).

VEGF positive (high immunoreactivity) tumours accounted for 104/223 (46.6%) cases examined and were associated with high vascular grade disease ($P = 0.009$) (Table 1). High PD-ECGF tumour cell immunoreactivity was seen in 72/223 (32.3%) cases and likewise correlated with angiogenesis ($P = 0.05$) (Table 1). There was a weak positive correlation between VEGF and PD-ECGF expression ($r = 0.21$, $P = 0.002$).

Eighteen of the 41 (44%) NSCLC tumours with high VEGF and PD-ECGF immunoreactivity had high vascular grade disease. This was equivalent to the proportion of VEGF only and PD-ECGF only positive tumours associated with high microvessel counts (31/63; 49% and 16/31; 48% respectively). As compared to angiogenic growth factor negative disease, positive tumours were strongly associated with high vascular grade (20/88: 22.7% vs 65/135: 48.1%; $P = 0.001$).

Although overall no association was found between VEGF and/or PD-ECGF expression and stage of disease, high PD-ECGF expression was seen more frequently in stage 1b as compared to stage 1a NSCLC tumours ($P = 0.02$).

### Survival analyses

Univariate analysis of survival showed that T-stage (T2 vs T1; $P = 0.01$: T3 vs T2 and T1; $P = 0.0001$), N-stage (N2 vs N1; $P = 0.02$: N2 vs N0; $P = 0.0001$: N1 vs N0; $P = 0.002$) and vascular grade (high vs low; $P = 0.0001$) were the most significant prognostic variables (Table 2) (Figure 1A). Both high tumour cell VEGF and PD-ECGF immunoreactivity were of prognostic significance ($P = 0.02$) (Table 2) (Figure 1B, C). Combined expression of VEGF and PD-ECGF did not identify a worse prognostic

© 2000 Cancer Research Campaign 

**Table 2** Univariate analysis of all variables analysed in the study

| Parameter | Hazard ratio | *P*-value |
|---|---|---|
| Histology | | |
| Adenocarcinoma vs squamous | 1.36 | 0.21 |
| T-stage | | |
| T3 vs T2 | 3.63 | 0.0001 |
| T3 vs T1 | 6.29 | 0.0001 |
| T2 vs T1 | 1.87 | 0.01 |
| N-stage | | |
| N2 vs N1 | 2.29 | 0.02 |
| N2 vs N0 | 4.15 | 0.0001 |
| N1 vs N0 | 1.95 | 0.002 |
| Grade | | |
| 3 vs 1/2 | 1.36 | 0.14 |
| Vascular grade | | |
| High vs low | 2.34 | 0.0001 |
| VEGF | | |
| Positive vs negative | 1.63 | 0.02 |
| PD-ECGF | | |
| Positive vs negative | 1.51 | 0.02 |

**Table 3a** Multivariate analysis of all tumour variables analysed in the study

| | Hazard ratio | | |
|---|---|---|---|
| Variable | t ratio | *P*-value | Significant? |
| Histology | 1.254 | 0.212 | No |
| T stage | 2.421 | 0.017 | Yes |
| N status | 1.509 | 0.133 | No |
| Grade | 0.8698 | 0.386 | No |
| CD31 | 2.080 | 0.039 | Yes |
| VEGF | 1.529 | 0.128 | No |
| PD-ECGF | 0.6769 | 0.499 | No |

**Table 3b** Multivariate analysis of variables significant at univariate analysis but VEGF/PD-ECGF considered as a single variable

| | Hazard ratio | | |
|---|---|---|---|
| Variable | t ratio | *P*-value | Significant? |
| T stage | 2.574 | 0.011 | Yes |
| N status | 1.504 | 0.134 | No |
| CD31 | 2.056 | 0.041 | Yes |
| VEGF/PD-ECGF | 1.941 | 0.054 | No |

subgroup of patients beyond that seen with VEGF or PD-ECGF alone (Figure 1D). T-stage ($P < 0.02$) and vascular grade ($P < 0.04$) were independent predictors of outcome on multivariate analysis when all parameters were considered (Table 3a). When analysed as a single variable, the presence of an angiogenic growth factor vs negative tumours approached significance in a multivariate model ($P = 0.054$) (Table 3b).

Although the numbers were relatively small the study included sufficient patients to analyse the impact of VEGF and PD-ECGF, and microvessel density on outcome in stage Ia to IIb disease (stage Ia, 37; stage Ib, 67; stage IIa, 19; and stage IIb, 44 patients). High vascular grade was associated with a poor prognosis in the stage IIb disease subgroup only ($P = 0.0005$). Over-expression of either or both of the angiogenic growth factors analysed was also associated with a poor outcome in stage IIb disease as compared to VEGF and PD-ECGF negative NSCLC ($P = 0.02$).

No association was found between histology and tumour grade and any of the parameters evaluated.

### DISCUSSION

The results support the findings of previous studies indicating that the intensity of intratumoural angiogenesis, as assessed by tumour microvessel counts, has prognostic significance and plays an important role in the pathogenesis of NSCLC (Giatromanolaki et al, 1996; Fontanini et al, 1997). This is in keeping with observations in other solid tumours including breast (Fox et al, 1995), colorectal (Amaya et al, 1997) and gastric cancer (Maeda et al, 1996; Takahashi et al, 1998).

It is important to note, however, that not all studies have found a positive association between microvessel density and prognosis (Apolinario et al, 1997; Chandrachud et al, 1997; Pezzella et al, 1997). Indeed in the largest of these studies tumours with an alveolar pattern, with no parenchymal destruction and the alveolar septae still present, had a worse outcome than those tumours with an angiogenic pattern. This observation indicates that if an appropriate blood supply is available, an NSCLC tumour may exploit it and grow within the lung without the need for neovascularization (Pezzella et al, 1997). The study was in stage I NSCLC only. This may, to some extent, account for the lack of an association between angiogenesis and outcome as high microvessel counts are strongly correlated with tumour size and nodal status as shown in the present study and our earlier work (Giatromanolaki et al, 1996). Finally, although there is intense debate on the subject (Fox et al, 1995), a recent study in lung tumours has shown considerable heterogeneity of vasculature in NSCLC specimens even within blocks from the same region of the tumour. Furthermore the periphery of the tumour, traditionally the area where blood vessels are counted, does not always contain the highest number of vessels (Schor et al, 1998).

A number of studies have demonstrated that VEGF expression is associated with angiogenesis and/or has prognostic significance in solid tumours including breast (Fox et al, 1995), colorectal (Amaya et al, 1997), gastic (Maeda et al, 1997; Takahashi et al, 1998) and pancreatic cancer (Ikeda et al, 1999). The results of the present work confirm previous findings of an important role for tumour cell VEGF expression in the angiogenic process of, and as a prognostic factor for, NSCLC (Volm et al, 1996; Giatromanolaki et al, 1998). VEGF overexpression was not associated with a particular stage of disease or with a negative outcome in any given disease stage subgroup (stage Ia to IIb). However, when all 183 patients were taken into consideration, univariate analysis revealed high VEGF immunoreactivity to be associated with a poor prognosis ($P = 0.02$). The VG1 monoclonal antibody employed in the current study detects the VEGF 121, 165 and 189 isoforms (Turley et al, 1998). The non-heparin binding VEGF 121 and the basic, heparin binding VEGF 165 are freely secreted from producing cells whilst the longer isoforms VEGF 189 and 206 are generally cell associated. Employing reverse transcription-polymerase chain reaction (RT-PCR) and in situ hybridization techniques, VEGF 121, VEGF 165 and VEGF 189 have been found to be associated with angiogenesis and/or a poor prognosis in NSCLC (Oshika et al, 1998; Fontanini et al, 1999).

The present study confirms our earlier work demonstrating that high PD-ECGF overexpression has a weak association with angiogenesis and prognosis, the latter on univariate analysis only. The findings are in keeping with the results of studies in malignant

© 2000 Cancer Research Campaign disease where PD-ECGF overexpression has been seen in a proportion of all solid tumours studied to date including breast (Toi et al, 1995), colorectal (Amaya et al, 1997), gastric (Maeda et al, 1997; Takahashi et al, 1998), oesophageal (Igarashi et al, 1998) and pancreatic (Ikeda et al, 1999) cancers. In general, PD-ECGF is associated with angiogenesis and/or advanced disease. Furthermore overexpression may predict for poor survival (Takebayashi et al, 1996; Ikeda et al, 1999). However, PD-ECGF immunoreactivity, be it in the malignant, inflammatory and/or stromal cells of the tumour, is not always associated with a poor outcome emphasizing the relatively weak prognostic power of this marker (Toi et al, 1995; Igarashi et al, 1998).

We observed a weak positive correlation between tumour VEGF and PD-ECGF expression ($r = 0.21$; $P = 0.002$). Similar observations have been made in colorectal cancer (Amaya et al, 1997). Although not seen in our study synergy between VEGF and PD-ECGF in determining the intensity of angiogenesis has been reported for breast, gastric and pancreatic cancer (Toi et al, 1995; Maeda et al, 1997; Ikeda et al, 1999). A correlation between tumour cell VEGF and PD-ECGF-positive CD68 infiltrating inflammatory cells has been observed in gastric cancer, the vessel count being significantly higher in tumours expressing both growth factors as compared to those expressing each growth factor alone (Takayashi et al, 1998). Furthermore, a significant association between co-expression of both angiogenic factors with the presence of hepatic metastases has been described in this disease (Maeda et al, 1997). We found that high immunoreactivity of either or both of the angiogenic growth factors analysed conferred a poor prognosis in stage IIb disease ($P = 0.02$). This may reflect the contribution of these growth factors to the angiogenic process which itself is associated with a particularly poor outcome in the stage IIb patients studied ($P = 0.0005$).

These findings indicate that VEGF may be a suitable target for novel therapies in the management of NSCLC. Inhibition of VEGF activity with monoclonal antibodies (Kim et al, 1993), soluble VEGF receptors (Lin et al, 1998) and VEGF-receptor 2 (Flk-1/KDR) monoclonal antibodies (Skobe et al, 1997) has shown promise in experimental in vivo models with inhibition of angiogenesis, tumour growth and/or tumour cell invasion being recorded.

As outlined earlier PD-ECGF is thymidine phosphorylase (TP), an important enzyme in the activation of 5-fluorouracil (5-FU) to fluorodeoxyuridine. TP also plays a role in converting 5-FU prodrugs, including 5′-deoxy-5-fluorouridine and the novel oral fluoropyrimidine carbamate, capecitabine, to their active metabolites (Patterson et al, 1995; Budman et al, 1998). A recent report by our group demonstrated that there was a significant improvement in both relapse-free and overall survival in PD-ECGF/TP-positive as compared with PD-ECGF/TP-negative breast cancer patients treated with adjuvant cyclophosphamide, methotrexate and 5-FU (CMF) chemotherapy. This was felt to be due not only to the activation of 5-FU but to the enhancement of the effectiveness of methotrexate by TP (Fox et al, 1997). Given the overexpression of PD-ECGF/TP seen in NSCLC, the combination of fluoropyrimidines with the more widely used cytotoxics, such as the platinums, taxanes, vinca alkaloids and topoisomerase I inhibitors, gemcitabine and ifosphamide, in the treatment of NSCLC should be evaluated further (van Zandwijk and Giaccone, 1996).

In the present study we have clearly demonstrated that a proportion of high vascular grade tumours do not express either VEGF or PD-ECGF indicating the importance of other angiogenic factors in this regard. Apart from the more established angiogenic growth factors such as VEGF and PD-ECGF recent evidence has suggested a role for tissue factor (TF) (Koomagi and Volm, 1998), heparin-binding growth-associated molecule (HB-GAM) (Jager et al, 1997) and CXC chemokines (Arenberg et al, 1997) in the induction of angiogenesis in NSCLC. As such other novel anti-angiogenic agents, including angiostatin, endostatin, TNP-470, thalidomide and the matrix metalloproteinase inhibitors (MMPIs) may have a role to play in the treatment of NSCLC (Harris, 1998; Macaulay et al, 1999).

In conclusion, VEGF and PD-ECGF are important angiogenic growth factors in NSCLC. The detection of a subset of high vascular grade tumours not dependent on either VEGF or PD-ECGF indicates that other angiogenic growth factors, such as bFGF, HB-GAM and TF, may have an important role in the pathogenesis of NSCLC.

### ACKNOWLEDGEMENTS

This work was supported by the Institute of Cancer Studies, Leicester and the Imperial Cancer Research Fund, UK and the Tumour and Angiogenesis Research Group, Greece.

### REFERENCES

Amaya H, Tanigawa N, Lu C, Matsumura M, Shimomatsuya T, Horiuchi T and Muraoka R (1997) Association of vascular endothelial growth factor expression with tumor angiogenesis, survival and thymidine phosphorylase/platelet derived endothelial cell growth factor expression in human colorectal cancer. *Cancer Lett* **119**: 227–235

Apolinario RM, van der Valk P, de Jong JS, Deville W, van Ark-Otte J, Dingemans A-MC, van Mourik JC, Postmus PE, Pinedo HM and Giaccone G (1997) Prognostic value of the expression of p53, bcl-2, and bax oncoproteins, and neovascularisation in patients with radically resected non-small cell lung cancer. *J Clin Oncol* **15**: 2456–2466

Arenberg DA, Polverini PJ, Kunkel SL, Shanafelt A, Hesselgesser J, Horuk R and Strieter RM (1997) The role of CXC chemokines in the regulation of angiogenesis in non-small cell lung cancer. *J Leukoc Biol* **62**: 554–562

Budman DR, Meropol NJ, Reigner B, Creaven PJ, Lichtman SM, Bergham E, Behr J, Gordon RJ, Osterwalder B and Griffin T (1998) Preliminary studies of a novel oral fluoropyrimidine carbamate: capecitabine. *J Clin Oncol* **16**: 1795–1802

Chandrachud LM, Pendleton N, Chisholm DM, Horan MA and Schor AM (1997) Relationship between vascularity, age and survival in non-small cell lung cancer. *Br J Cancer* **76**: 1367–1375

Ferrara N and Davis-Smyth T (1997) The biology of vascular endothelial growth factor. *Endoc Rev* **18**: 4–25

Fontanini G, Lucchi M, Vignati S, Mussi A, Ciardiello F, De Laurentiss M, De Placido S, Basolo F, Angeletti CA and Bevilacqua G (1997) Angiogenesis as a prognostic indicator of survival in non-small cell lung carcinoma: a prospective study. *J Natl Cancer Inst* **89**: 881–886

Fontanini G, Boldrini L, Chine S, Pisaturo F, Basolo F, Calcinai A, Lucchi M, Mussi A, Angeletti CA and Bevilacqua G (1999) Expression of vascular endothelial growth factor mRNA in non-small cell lung carcinomas. *Br J Cancer* **79**: 363–369

Fox SB, Leek RD, Weekes MP, Whitehouse RM, Gatter KC and Harris AL (1995) Quantification and prognostic value of breast cancer angiogenesis: comparison of microvessel density, Chalkley count and computer image analysis. *J Pathol* **177**: 275–283

Fox SB, Engels K, Comley M, Whitehouse RM, Turley H, Gatter KC and Harris AL (1997) Relationship of elevated tumour thymidine phosphorylase in node-positive breast carcinomas to the effects of adjuvant CMF. *Ann Oncol* **8**: 271–275

Giatromanolaki A, Koukourakis M, O'Byrne K, Fox S, Whitehouse R, Talbot DC, Harris AL and Gatter K (1996) Prognostic value of angiogenesis in operable non-small cell lung cancer. *J Pathol* **179**: 80–88

Giatromanolaki A, Koukourakis M, Comley M, Kaklamanis L, Turley H, O'Byrne K, Harris AL and Gatter K (1997) Platelet derived endothelial cell growth

© 2000 Cancer Research Campaign factor (thymidine phosphorylase) expression in lung cancer. *J Pathol* **181**: 196–199
Giatromanolaki A, Koukourakis MI, Kakolyris S, Turley H, O'Byrne KJ, Scott PAE, Pezzella F, Georgoulias V, Harris AL and Gatter KC (1998) Vascular endothelial growth factor, wild-type p53 and angiogenesis in early operable non-small cell lung cancer. *Clin Cancer Res* **4**: 3017–3024
Harris AL (1998) Are angiostatin and endostatin cures for cancer? Commentary. *Lancet* **351**: 1598–1599
Ikeda N, Adachi M, Taki T, Huang C, Hashida H, Takabayashi A, Sho M, Nakajima Y, Kanehiro H, Hisanaga M, Nakano H and Miyaka M (1999) Prognostic significance of angiogenesis in human pancreatic cancer. *Br J Cancer* **79**: 1553–1563
Igarashi M, Dhar DK, Kubota H, Yamamoto A, El-Assal O and Nagasue N (1998) The prognostic significance of microvessel density and thymidine phosphorylase expression in squamous cell carcinoma of the esophagus. *Cancer* **82**: 1225–1232
Ishikawa F, Miyazono K, Hellman U, Dexler H, Wernstedt C, Hagiwara K, Usuki K, Takaku F, Risau W and Heldin CH (1989) Identification of angiogenic activity and the cloning and expression of platelet-derived endothelial cell growth factor. *Nature* **338**: 557–562
Jager R, Noll K, Havemann K, Pfluger KH, Knabbe C, Rauvala H and Zugmaier G (1997) Differential expression and biological activity of the heparin-binding growth-associated molecule (HB-GAM) in lung cancer cell lines. *Int J Cancer* **73**: 537–543
Kim KJ, Li B, Winer J, Armanini M, Gillett N, Phillips HS and Ferrara N (1993) Inhibition of vascular endothelial growth factor-induced angiogenesis suppresses tumour growth in vivo. *Nature* **362**: 841–844
Koomagi R and Volm M (1998) Tissue-factor expression in human non-small-cell lung carcinoma measured by immunohistochemistry: correlation between tissue factor and angiogenesis. *Int J Cancer* **79**: 19–22
Koukourakis MI, Giatromanolaki A, Kakolyris S, O'Byrne KJ, Apostolikas N, Skarlatos J, Gatter KC and Harris AL (1998) Different pattern of stromal and cancer cell thymidine phosphorylase reactivity in non small cell lung cancer. Impact on tumour neoangiogenesis and survival. *Br J Cancer* **77**: 1696–1703
Lin P, Sanka S, Shan S, Dewhirst MW, Ploverini PJ, Quinn TQ and Peters KG (1998) Inhibition of tumor growth by targeting tumor endothelium using a soluble vascular endothelial growth factor receptor. *Cell Growth Diff* **9**: 49–58
Maeda K, Kang SM, Ogawa M, Onoda N, Sawada T, Nakata B, Kato Y, Chung YS and Sowa M (1997) Combined analysis of vascular endothelial growth factor and platelet-derived endothelial cell growth factor expression in gastric carcinoma. *Int J Cancer* **74**: 545–550
Macaulay VM, O'Byrne KJ, Saunders MP, Long L, Gleeson F, Puttick R, Harris AL and Talbot DC (1999) Phase I study of intrapleural batimastat (BB-94), a matrix metalloproteinase inhibitor, in the treatment of malignant pleural effusions. *Clin Cancer Res* **5**: 513–520
Moghaddam A and Bicknell R (1992) Expression of platelet-derived endothelial cell growth factor in *Escherichia coli* and confirmation of its thymidine phosphorylase activity. *Biochemistry* **31**: 12141–12146
Moghaddam A, Zhang HT, Fan TPD, Hu D-E, Lees V, Turley H, Fox SB, Gatter KC, Harris AL and Bicknell R (1995) Thymidine phosphorylase is angiogenic and promotes tumour growth. *Proc Natl Acad Sci USA* **92**: 998–1002
Oshika Y, Nakamura M, Tokunaga T, Ozeki Y, Fukushima Y, Hatanaka H, Abe Y, Yamazaki H, Kijima H, Tamaoki N and Ueyama Y (1998) Expression of cell-associated isoform of vascular endothelial growth factor 189 and its prognostic relevance in non-small cell lung cancer. *Int J Oncol* **12**: 541–544
Patterson AV, Zhang H, Moghaddam A, Bicknell R, Talbot DC, Stratford IJ and Harris AL (1995) Increased sensitivity to the prodrug 5′-deoxy-5-fluorouridine and modulation of 5-fluoro-2′-deoxyuridine sensitivity in MCF-7 cells transfected with thymidine phosphorylase. *Br J Cancer* **72**: 669–675
Pezzella F, Pastorino U, Tagliabue E, Andreola S, Sozzi G, Gasparini G, Menard S, Gatter KC, Harris AL, Fox S, Buyse M, Pilotti S, Pierotti M and Rilke F (1997) Non-small-cell lung carcinoma tumor growth without morphological evidence of neo-angiogenesis. *Am J Pathol* **151**: 1417–1423
Schor AM, Pazouki S, Morris J, Smither RL, Chandrachud LM and Pendleton N (1998) Heterogeneity in microvascular density in lung tumours: comparison with normal bronchus. *Br J Cancer* **77**: 946–951
Skobe M, Rockwell P, Goldstein N, Vosseler S and Fusenig NE (1997) Halting angiogenesis suppresses carcinoma cell invasion. *Nat Med* **3**: 1222–1227
Takahashi Y, Bucana CD, Akagi Y, Liu W, Cleary KR, Mai M and Ellis LM (1998) Significance of platelet-derived endothelial cell growth factor in the angiogenesis of human gastric cancer. *Clin Cancer Res* **4**: 429–434
Takebayashi Y, Akiyama S, Akiba S, Yamada K, Miyadera K, Sumizawa T, Yamada Y, Murata F and Aikou T (1996) Clinicopathologic and prognostic significance of an angiogenic factor, thymidine phosphorylase, in human colorectal carcinoma. *J Natl Cancer Inst* **88**: 1110–1117
Toi M, Hosina S, Taniguchi T, Yamamoto Y, Ishitsuka H and Tominaga T (1995) Vascular endothelial growth factor and platelet derived endothelial cell growth factor are frequently coexpressed in highly vascularized human breast cancer. *Clin Cancer Res* **1**: 961–964
Turley H, Scott PAE, Watts VM, Bicknall R, Harris AL and Gatter KC (1998) Expression of VEGF in routinely fixed material using a new monoclonal antibody VG1. *J Pathol* **186**: 313–318
van Zandwijk N and Giaccone G 1996 Treatment of metastatic non-small cell lung cancer. *Curr Opin Oncol* **8**: 120–125
Volm M, Koomagi R and Mattern J (1996) Interrelationships between microvessel density, expression of VEGF and resistance to doxoresistance of non-small-cell lung carcinoma. *Anticancer Res* **16**: 213–218
Whittle C, Gillespie K, Harrison R, Mathieson PW and Harper SJ (1999) Heterogeneous vascular endothelial growth factor (VEGF) isoform mRNA and receptor mRNA expression in human glomeruli, and the identification of VEGF148 mRNA, a novel truncated splice variant. *Clin Sci (Colch)* **97**: 303–312

 *© 2000 Cancer Research Campaign*
